# Mortality ascertainment of participants in the National Wilms Tumor Study using the National Death Index: comparison of active and passive follow-up results

**DOI:** 10.1186/1742-5573-4-5

**Published:** 2007-07-02

**Authors:** Cecilia A Cotton, Susan Peterson, Patricia A Norkool, Norman E Breslow

**Affiliations:** 1 Department of Biostatistics, University of Washington, Box 357232, Seattle, WA 98195, USA; 2 National Wilms Tumor Study, Fred Hutchinson Cancer Research Center, 1100 Fairview Ave N, M2-A876, PO Box 19024, Seattle, WA 98109, USA

## Abstract

Long term studies of childhood cancer survivors are hampered by difficulties in tracking young adult participants. After performing a National Death Index (NDI) search we sought to identify which factors best predicted a match among known decedents from the National Wilms Tumor Study (NWTS) and to determine if record linkage could substitute for missing follow-up in a cohort of NWTS survivors. To our knowledge, this is the first study to compare passive mortality follow-up using the NDI to active follow-up of a childhood and young adult population.

Records for 984 known decedents and 3,406 subjects whose January 1, 2002 vital status was unknown were sent to the NDI in June 2003. In April 2005 NWTS follow-up records were used to reassess January 1, 2002 vital status. Matches were established for 709 of 789 known decedents (sensitivity 89.9%) with a date of death between 1979 and 2001, the calendar period covered by the NDI at the time of the search. No matches were identified among 1,052 subjects known to be alive in 2002 (specificity 100%). Factors associated with decreased sensitivity were an unknown social security number (sensitivity 87.8%), Hispanic ethnicity (76.4%) and foreign birth (56.5%). For 2,351 subjects with 2002 vital status unknown who had 13,166 pre 2002 person-years of missing observation, only 18 deaths were ascertained by the NDI whereas 79.3 were expected based on NWTS mortality data. Mortality analyses based strictly on NDI search results and those based on NWTS follow-up augmented with NDI search results yielded inflated estimates of the 15 year survival rate when compared with estimates based on NWTS active follow-up.

Match rates were comparable to those observed in adult populations. Since the same selection factors were likely associated with NDI failure to match and NWTS loss to follow-up, use of the NDI to fill in missing follow-up data appears unwarranted.

## Introduction

The remarkable progress in treatment of childhood cancer during the past few decades has focused attention on the health status of the growing population of survivors [1-3]. Such research is hampered, however, by difficulties in locating teenage and adult survivors and by their reluctance to return to pediatric hospitals [4]. Studies that actively follow participants by repeated contact are subject to drop-out that may differentially affect particular subgroups, e.g., underprivileged minorities [5]. Although statistical methods adjust for such loss in estimating population mortality rates, serious bias may result if subjects who drop out differ in health status from those who remain on study [6,7]. Studies that maintain passive follow-up by linkage of clinical records to disease or mortality registries are likewise subject to bias from failure of matching algorithms or events that occur outside the jurisdiction of the registry [8].

The National Death Index (NDI) is a centralized registry maintained by the National Center for Health Statistics of all deaths that occurred in the United States, Puerto Rico, and the Virgin Islands since 1979 [9]. Record linkage through the NDI has been well tested in adult populations [8] and is now commonly used to study death rates in adult cohorts [10-14]. The NDI has also been used to ascertain mortality in cohorts of children and young adults [15-17] but a literature search revealed no systematic evaluation of its use in these populations. In addition, no previous work was found evaluating the NDI as a means of augmenting follow-up in a study that attempts to maintain active contact with all participants.

Our study compared mortality ascertainment via active follow-up of a large cohort of subjects with childhood kidney cancer to mortality ascertainment using record linkage to the NDI. There were two specific aims: first, to identify which factors best predicted an NDI match among known decedents; and second, to determine how well information from the NDI search might substitute for missing follow-up data for those alive at last contact, but lost to follow-up before the closing date, in an attempt to improve the accuracy of mortality estimates.

## Analysis

### Study population

Between 1969 and 1995, medical institutions in the US and Canada enrolled 6,618 children with renal neoplasms on one of four randomized clinical trials conducted by the National Wilms Tumor Study (NWTS) Group [18-21]. Nearly 95 percent of enrollees had Wilms tumor while the remainder had clear cell sarcoma (CCSK), rhabdoid tumor of the kidney (RTK) or other rare histologic variants [22]. From the outset the NWTS emphasized continued follow-up of surviving participants as part of a late effects study to increase understanding of the long-term effects of diagnosis and treatment on development of second malignant neoplasms, congestive heart failure, renal failure and adverse pregnancy outcomes [23-26].

Follow-up is rigorously pursued for all on-study participants. Data requests are sent annually to the institution where the subject received treatment, for subjects still returning there, or otherwise directly to participants or a designated family member. When the most recent record for any subject is more than two years out-of-date, the computer system flags the case for immediate action. If the subject is not returning to the institution for follow-up, the NWTS requests authorization to track the participant in order to reestablish annual contact directly. If the NWTS discovers that a participant already in direct contact has moved, aggressive tracking is initiated to locate and renew contact with the participant.

The cohort for the present investigation comprised the 6,217 subjects enrolled from US institutions. By June, 2003, 198 were known to have died prior to January 1, 1979; 786 were known to have died after 1978 but prior to January 1, 2002, the closing date of the study; 1,827 were known to be alive on the closing date; and 3,406 had vital status unknown. Available identifying information for the 984 known decedents and for the 3,406 with vital status unknown was submitted to the NDI, which at the time of submission in June 2003 had collected data on deaths occurring in the US from 1979 through 2001.

The NWTS clinical trial protocols were approved by the Institutional Review Board (IRB) of the Fred Hutchinson Cancer Research Center and by the IRB of each participating institution. Each subject, or in the vast majority of cases their parent/guardian, provided informed consent at the time of enrollment. At age 18 each subject was contacted and asked to provide their own informed consent for continued participation as an adult.

### National death index search

Details of the NDI matching procedure have been discussed in detail elsewhere [9,27]. Multiple potential NDI record matches are frequently identified for a single submitted user record. It is the investigator's responsibility to assess match quality and to make the final determination of match status. Briefly, in the cohort of known decedents all exact matches, where the NDI and NWTS records matched on all available identifiers, were accepted. For the remaining decedents and for subjects with vital status unknown potential matches assigned a suitably high score by the NDI [27] or which we determined to be of interest were assessed manually. This involved searching the subject's chart for information bearing on the validity of the potential match. For example, we compared the NDI recorded state of death with the state(s) where the subject resided or was treated and checked for coding errors that might explain discrepancies in date of birth or SSN.

### Statistical analysis

Vital status on January 1, 2002 was updated from NWTS follow-up records in April, 2005. The updated cohort of subjects known to have died between 1979 and 2001 was used to determine which of the following factors were associated with an NDI match: sex, ethnicity (non-Hispanic Caucasian, African American, Asian, Hispanic, unknown/other), Social Security Number (SSN) on file, year of death, country of birth (United States (US), foreign or unknown), age at death, geographic location of treatment institution (northern border, southern border, non-border state), institutional compliance (the fraction of cases from the registering institution in active follow-up) and NWTS recorded cause of death (tumor, toxicity or infection, toxicity with viable tumor present, other/unknown). Each factor was examined separately and then all were examined together using multiple logistic regression. Records submitted to the NDI due to unknown survival status as of June 2003, but which when updated in 2005 showed survival beyond that date, were used to assess the specificity of the NDI matches.

Survival estimates based on information acquired through standard NWTS follow-up procedures were compared with those based on NWTS follow-up augmented with results from the NDI search. All analyses used a closing date of January 1, 2002. Deaths prior to 1979 were included in the analyses so as to be consistent with other reports from the NWTS and provide accurate overall survival estimates. All survival analyses were stratified by NWTS study number into the "early" era (NWTS-1,2) and "modern" era (NWTS-3,4) to account for changes in survival which occurred mainly due to substantial therapeutic advances made over the course of the clinical trials. For the standard analysis, which used only the updated 2005 NWTS information, subjects lost to follow-up before the closing date had their records censored. For the augmented analysis, subjects last known alive prior to closing who had no NDI match had their date last seen updated to January 1, 2002, whereas those who had a match were considered to have died on the date supplied by the NDI.

Because we did not submit the entire NWTS cohort to the NDI we could not directly compare the results of a mortality study using an NDI search alone with mortality based on active follow-up through the NWTS. As a representation of what might have happened had the NWTS halted follow-up and relied solely on the NDI results, a third analysis was preformed in which subjects known to the NWTS to have died after 1978 but who were not matched through the NDI were considered to have been alive on January 1, 2002. For all analyses, overall survival percentages and 95 percent Confidence Intervals (CIs) were estimated at 15 years from diagnosis by actuarial methods [7,28].

Actuarial methods were also used to calculate 10 year percentages and 95% CIs of loss to follow-up by the NWTS among subjects enrolled prior to January 1, 1990. The policy of the NWTS is to start tracking procedures once a subject has been out of contact for two years. The selection of a cut-off date twelve years prior to the study closing date allows each subject to have had the potential for at least ten years of follow-up.

To determine how well passive follow-up through the NDI could substitute for active follow-up, the number of deaths ascertained through the NDI search was compared with an expected number based on NWTS mortality rates. The entire cohort of 6,217 subjects was used to determine numbers of deaths and person-years of follow-up by age, time since diagnosis, gender, study number (NWTS-1-4), stage of disease (I-V), histology (favorable or anaplastic Wilms tumor, CCSK, RTK or other) and the availability of an SSN. The Lexis package [29] developed for the R statistical [30] was used to form the seven dimensional tables. Mortality rates were calculated by dividing numbers of deaths by person-years of follow-up in each cell of the table. For subjects who had their follow-up augmented by the NDI, i.e. those who in June 2005 were still last known to be alive prior to closing and for whom no NDI match was found, a similar table was constructed of augmented person-years at risk from the later of date last seen or January 1, 1979 to closing. Multiplying the mortality rates for the entire cohort by the augmented person-years gave the expected number of deaths in each cell. Observed and expected mortality rates for this period of augmented follow-up were compared by availability of a SSN and other factors. Poisson based p-values for testing whether the observed number of deaths could be adequately explained by expected mortality rates were calculated using Byar's approximation as described by Breslow and Day [31].

## Results

**Figure 1:**
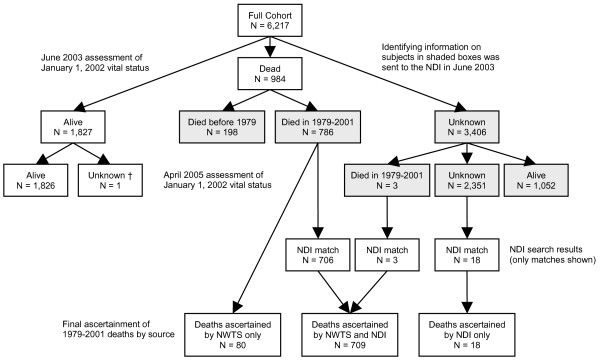
Breakdown of NWTS* cohort by January 1, 2002 vital status and NDI* match status. * NWTS, National Wilms Tumor Study; NDI, National Death Index. † Record not sent to the NDI due to an error in date last seen which was later corrected.

A total of 706 NDI matches were established among the 786 subjects known as of June, 2003 to have died between 1979 and 2001. No NDI matches were identified in the group of known decedents with deaths prior to 1979. A further 21 matches were identified from the cohort of 3,406 NWTS subjects with vital status unknown on January 1, 2002. Three of these deaths were also ascertained in April 2005 when NTWS follow-up was used to update records of the 3,406 subjects whose vital status was unknown at the time of the NDI submission. Thus the NDI record search matched 709 of the 789 deaths (89.8 percent) known by the NWTS to have occurred between 1979 and closing. After the 2005 update, 1,052 subjects with vital status previously unknown were found to have survived into 2002 or beyond. No NDI matches were identified among these 1,052 subjects giving a match specificity of 100 percent. A full breakdown of the cohort by January 1, 2002 vital status as assessed in June 2003 and again in April 2005, with ascertainment of deaths by the NDI and the NWTS is given in Figure 1. A further cross-tabulation of NDI match status by January 1, 2002 vital status is shown in Table 1. The individual with unknown vital status whose record was not sent to the NDI had a recorded date last seen that changed from after January 1, 2002 to before that date during the mortality update.

**Table 1:** NDI* match counts by January 1, 2002 vital status as ascertained in April 2005

	January 1, 2002 vital status ascertained in April, 2005 from NWTS* records									
	Dead				Alive		Unknown		Total (%)	
NDI*	1970–1978		1979–2001							
Match	0		709		0		18		727	(11.7)
No Match	198		80		1,052		2,333		3,663	(58.9)
Not sent	0		0		1,826		1†		1,827	(29.4)
Total (%)	198	(3.2)	789	(12.7)	2,878	(46.3)	2,352	(37.8)	6,217	(100.0)

* NDI, National Death Index; NWTS, National Wilms Tumor Study.

† Record not sent to the NDI due to an error in date last seen which was later corrected

Table 2 shows match rates for the 789 known decedents. Using multiple logistic regression, three factors were independently associated with a lowered match rate: lack of SSN, Hispanic ethnicity and birth outside the US. Sensitivity was 96.7 percent for subjects with an SSN on file but only 87.8 percent for those without. Sensitivities were also substantially lower for Hispanics (76.4 percent) and for those born abroad (56.5 percent) compared to those of non-Hispanic Caucasians and US born, respectively. Subjects who died during earlier calendar periods were also less likely to have a match, but year of death was not a statistically significant factor after adjustment for the confounding effects of SSN. Age at death was not significantly associated with the probability of having a match. This was true when age at death was included as a continuous variable (p = 0.16) or as a grouped linear variable (p = 0.08).

**Table 2:** Demographic characteristics and NDI* match counts of the known decedents (1979–2001) as ascertained in April 2005

	Total	Match	% Match	OR*†	95% CI*†
Sex					
Female	413	364	88.1	1.00	
Male	376	345	91.8	1.49	(0.90, 2.48)
Ethnicity					
Caucasian, non-Hispanic	551	507	92.0	1.00	
African American	147	131	89.1	0.67	(0.35, 1.25)
Asian	13	12	92.3	1.19	(0.13, 11.10)
Hispanic	72	55	76.4	0.30	(0.14, 0.63)
Unknown/Other	6	4	66.7	0.31	(0.05, 1.99)
SSN					
Unknown	606	532	87.8	1.00	
Known	183	177	96.7	4.04	(1.54, 10.58)
Place of birth					
United States	623	568	91.2	1.00	
Foreign	23	13	56.5	0.12	(0.04, 0.34)
Unknown	143	128	89.5	1.02	(0.54, 1.94)
Age at death					
0–4 years	245	231	90.9	1.00	
5–9 years	329	291	88.4	0.61	(0.33, 1.10)
10–19 years	156	141	90.4	0.60	(0.28, 1.32)
20+ years	50	46	92.0	0.35	(0.09, 1.37)
Total	789	709	89.9		

* NDI, National Death Index; OR, odds ratio; CI confidence interval.

† Odds ratios and 95 percent confidence intervals adjusted for sex, ethnicity, presence of SSN, year of death, location of birth, age at death group, location of treatment institution, institutional compliance and NWTS recorded cause of death.

Results of the survival analyses comparing standard NWTS, NDI supplemented and NDI only follow-up are presented in Table 3. These are presented separately for the "early" (NWTS-1,2) and "modern" (NWTS-3,4) treatment eras. After augmenting the NWTS data with NDI search results, 2,351 subjects with vital status unknown on closing had an average of 5.6 years of follow-up added to their records between 1979 and 2001 for a total of 13,166 person-years. Eighteen deaths based on the NDI match were added to the 198 pre- and 789 post-1979 deaths ascertained by the NWTS. The NDI augmentation led to small increases in each of the actuarial survival estimates.

**Table 3:** Estimated survival at 15 years from diagnosis calculated using follow-up from NWTS* records only†, NWTS records supplemented with NDI* search results and NDI search results only

		NWTS only			NWTS + NDI			NDI only		
	Total	Died	% Alive	95% CI*	Died	% Alive	95% CI	Died	% Alive	95% CI
NWTS-1,2	1,310	317	77.8	(75.4, 79.9)	322	78.1	(75.8, 80.2)	307	79.0	(76.7, 81.1)
NWTS-3,4	4,907	670	85.7	(84.6, 86.7)	683	86.0	(85.0, 87.0)	618	87.4	(86.4, 88.3)

* NWTS, National Wilms Tumor Study; NDI, National Death Index; CI, confidence interval.

† April 2005 ascertainment of January 1, 2002 vital status.

Since the actuarial procedure for the augmented data increased the date last seen to January 1, 2002 for all surviving subjects, but increased the numbers of deaths only by those that occurred after 1978, part of the apparent increase in survival was due to failure to account for deaths before 1979 in subjects lost to follow-up earlier. However, only 43 person-years of follow-up, during which 2.3 deaths would have been expected, were added to the pre-1979 period as a result of the NDI search. Consequently, any bias caused by the gap before the NDI became operational would be minimal.

The NDI only analysis removed 80 deaths, added in a total of 1,170 person-years of follow-up for these 80 subjects and resulted in additional increases in the survival estimates. The improvement in survival from the first two NWTS trials to the second two is apparent no matter which death ascertainment method is used.

Among the 2,351 subjects whose follow-up was augmented by NDI search data we would have expected 79.3 deaths between 1979 and 2001 based on NWTS mortality rates. This is 4.4 fold higher than the 18 additional deaths actually found. SSNs were available for 23 percent of known decedents and 58 percent of subjects with an unknown vital status. The NDI search identified 6/12.1 (50 percent) of the deaths expected among participants with an SSN but only 12/67.3 (18 percent) of the deaths expected among those without. The difference between observed and expected deaths was not statistically significant among participants with an SSN (p = 0.09) but was among those without an SSN (p < 0.001).

Participants of non-Hispanic Caucasian ethnicity and those born in the United States had much better follow-up rates than children of other ethnicities and places of birth. By 10 years after enrollment 9.5 percent [95% CI: (8.5, 10.7)] of non-Hispanic Caucasian participants were lost to follow-up while 22.4 percent (19.5, 25.8) of African American, 19.5 percent (10.2, 35.4) of Asian and 25.9 percent (21.2, 31.4) of Hispanic participants had been lost. Of participants born in the US, 12.2 percent (11.1, 13.3) were lost by 10 years as compared to 35.5 percent (24.8, 49.2) of foreign born participants. No significant difference was seen across gender. Only 148 (6.1 percent) of the 2417 children with an SSN on file at the time of the NDI submission were lost to follow-up before 10 years, whereas 362 (17.2 percent) of the 2,105 without an SSN were lost. Since the date of SSN acquisition was not recorded, however, this precluded proper assessment of its effect as a time-dependent covariate on reducing loss to follow-up.

## Conclusion

Three factors were independently associated with a lowered NDI match rate among known decedents: lack of SSN, Hispanic ethnicity and birth outside the US. Using NWTS mortality rates we estimated that 79.3 deaths should have occurred among subjects lost to follow-up yet only 18 such deaths were ascertained by the NDI search. We conclude that, in populations such as ours that are actively followed, the NDI cannot be used to reliably fill in missing follow-up data and that doing so may lead to inflated survival estimates.

To the best of our knowledge this was the first study designed to evaluate the NDI as a means of substituting for follow-up data in children and young adults. In the NWTS cohort, over 70 percent of deaths occurred before age 10. The bulk of missing follow-up time was while subjects were between 10 and 30 years of age. Several previous studies have used the NDI in similar populations but either lacked their own follow-up data or did not use it as a means of assessing the NDI [15-17,32].

The sensitivities of an NDI match observed for subjects with and without an SSN on file, 97 percent and 88 percent, are within the range of match rates found in previous studies of adults [8]. During the early years of the study parents were unlikely to have applied for SSNs for their young children. SSNs were not routinely requested for subjects on the NWTS late effects and therapeutic studies until 1979 and 1986, respectively. Our finding that children with an SSN on file had lower rates of loss is in part a statistical artifact, caused by the fact that SSNs were requested by parents and ascertained by the NWTS throughout the study, so those with longer periods of follow-up would have had more opportunity to get an SSN. Parents or adult subjects who report SSNs may also be easier to track for other reasons. Nonetheless, the message is clear that studies with a mortality end-point that anticipate long follow-up periods or a high potential for study drop out should endeavor to collect SSNs both to facilitate tracking and to increase the effectiveness of any future NDI search.

One recent study based on active follow-up of Hispanics over age 65 suggested that NDI linkage rates may under-ascertain deaths in this population [33]. While the results of this study are under review and may have been overstated [34] , our results demonstrate the same phenomenon for young Hispanic subjects. A small portion of the under-ascertainment in adults has been attributed to "salmon bias", the likelihood that foreign born subjects will return home to their country of origin once they become ill [33]. A small portion of the under-ascertainment in our pediatric population was likewise attributable to the low (56.5 percent) match sensitivity observed for subjects known to be foreign born (n = 23), many of whom presumably returned home once treatment was completed.

Dividing 18 by 79.3 gives an expected match rate (22.7 percent) for the presumably deceased subjects that is much lower than the match rate observed for known decedents (89.9 percent). Part of this discrepancy could be due to bias in NWTS rates, for example, the "bad news travels fast" principle such that deaths are reported in preference to the fact that the subject is alive and well. In this case NWTS death rates would be overestimated. Most of the discrepancy, however, is likely due to very substantial under-ascertainment by the NDI of the deaths that presumably occurred in this group of subjects during the period they were lost to follow-up.

Factors associated with a loss of contact by the NWTS, such as emigration from the US, are also associated with a lower probability of an NDI match in case of death. In other words, dropout is subject to the same selection bias factors as the failure of the NDI to match a death. This was demonstrated here for two factors on which information was available: Hispanic ethnicity and foreign birth. It is undoubtedly true also for other non measured and intangible factors. The results of our analysis suggest that relying solely on NDI search results in this situation would bias survival estimates upward.

This study serves as a reminder that subjects who are lost to follow-up are highly selected on factors related to our ability to maintain contact with them. Whether or not they are also selected on the factor that counts most, their likelihood of survival relative to that of subjects who remain in follow-up, is in principle unknowable without a perfect ascertainment system. While an NDI search can often substitute for extensive and expensive mortality follow-up, studies that actively maintain their own reasonably good contacts with participants may not benefit from using the NDI to substitute for follow-up in subjects with whom they have lost contact. The cost of performing an NDI search may not be justifiable. Nonetheless, our study will benefit substantially from the NDI search since we employed the optional NDI Plus service that provides the cause of death codes found on death certifications supplied to the NDI by the states. This greatly reduced the work required to ascertain official causes of death since death certificates for unmatched decedents must be requested directly from the states. Work is currently in progress on a comprehensive analysis of mortality in survivors of Wilms tumor that will include a comparison of cause-specific death rates to those in the general population.

## List of Abbreviations used

CCSK Clear Cell Sarcoma

CI Confidence Interval

IRB Institutional Review Board

NDI National Death Index

NWTS National Wilms Tumor Study

OR Odds Ratio

RTK Rhabdoid Tumor of the Kidney

SSN Social Security Number

US United States

## Competing interests

The author(s) declare that they have no competing interests.

## Authors' contributions

CAC carried out the statistical analyses and drafted the manuscript. SP participated in study design and contributed to the collection and validation of the data. PAK contributed to the collection and validation of the data and helped draft the manuscript. NEB conceived and designed the study, provided guidance for the analyses and helped draft the manuscript. All authors read and approved the final manuscript.

## References

[B1] Gurney JG, Davis S, Severson RK, Fang JY, Ross JA, Robison LL (1996). Trends in cancer incidence among children in the US. Cancer.

[B2] Ries LAG, Smith MA, Gurney JG, Linet M, Tamra T, Young JL, Bunin GR (1999). Cancer incidence and survival among children and adolescents: United States SEER program 1975–1995.

[B3] Robison LL, Mertens AC, Boice JD, Breslow NE, Donaldson SS, Green DM, Li FP, Meadows AT, Mulvihill JJ, Neglia JP, Nesbit ME, Packer RJ, Potter JD, Sklar CA, Smith MA, Stovall M, Strong LC, Yasui Y, Zeltzer LK (2002). Study design and cohort characteristics of the childhood cancer survivor study: A multi-institutional collaborative project. Med Pediatr Oncol.

[B4] Oeffinger KC, Eshelman DA, Tomlinson GE, Buchanan GR (1998). Programs for adult survivors of childhood cancer. J Clin Oncol.

[B5] Breslow N, Olshan A, Beckwith JB, Moksness J, Feigl P, Green D (1994). Ethnic variation in the incidence, diagnosis, prognosis, and follow-up of children with Wilms' tumor. J Natl Cancer Inst.

[B6] Fisher L, Kanarek P, Proschan F, Serfling RJ (1974). Presenting censored survival data when censoring and survival times may not be independent. Reliability and Biometry.

[B7] Kaplan EL, Meier P (1958). Nonparametric estimation from incomplete observations. J Am Stat Assoc.

[B8] Cowper DC, Kubal JD, Maynard C, Hynes DM (2002). A primer and comparative review of major US mortality databases. Ann Epidemiol.

[B9] National Center for Health Statistics (2000). National Death Index user's manual.

[B10] Marcus PM, Bergstralh EJ, Fagerstrom RM, Williams DE, Fontana R, Taylor WF, Prorok PC (2000). Lung cancer mortality in the Mayo Lung Project: impact of extended follow-up. J Natl Cancer Inst.

[B11] Greenfield TK, Rehm J, Rogers JD (2002). Effects of depression and social integration on the relationship between alcohol consumption and all-cause mortality. Addiction.

[B12] Rana JS, Mukamal KJ, Morgan JP, Muller JE, Mittleman MA (2004). Obesity and the risk of death after acute myocardial infarction. Am Heart J.

[B13] Rauscher GH, Sandler DO (2005). Validating Cancer Histories in Deceased Relatives. Epidemiology.

[B14] Sorlie PD, Coady S, Lin C, Arias E (2004). Factors Associated with Out-of-Hospital Coronary Heart Disease Death: The National Longitudinal Mortality Study. Ann Epidemiol.

[B15] Decoufle P, Autry A (2002). Increased mortality in children and adolescents with developmental disabilities. Paediatr Perinat Epidemiol.

[B16] Mills JL, Schonberger LB, Wysowski DK, Brown P, Durako SJ, Cox C, Kong F, Fradkin JE (2004). Long-term mortality in the United States cohort of pituitary-derived growth hormone recipients. J Pediatr.

[B17] Carroll-Pankhurst C, Engels EA, Strickler HD, Goedert JJ, Wagner J, Mortimer EA (2001). Thirty-five year mortality following receipt of SV40- contaminated polio vaccine during the neonatal period. Br J Cancer.

[B18] D'Angio GJ, Breslow N, Beckwith JB, Evans A, Baum E, deLorimier A, Fernbach D, Hrabovsky E, Jones B, Kelalis P, Otherson HB, Tefft M (1989). Treatment of Wilms' tumor. Results of the Third National Wilms' Tumor Study. Cancer.

[B19] D'Angio GJ, Evans A, Breslow N, Beckwith B, Bishop H, Farewell V, Goodwin W, Leape L, Palmer N, Sinks L, Sutow W, Tefft M, Wolff J (1981). The treatment of Wilms' Tumor: results of the Second National Wilms' Tumor Study. Cancer.

[B20] D'Angio GJ, Evans AE, Breslow N, Beckwith B, Bishop H, Feigl P, Goodwin W, Leape LL, Sinks LF, Sutow W, Tefft M, Wolff J (1976). The treatment of Wilms' tumor: Results of the National Wilms' Tumor Study. Cancer.

[B21] Green DM, Breslow NE, Beckwith JB, Finklestein JZ, Grundy RE, Thomas PR, Kim T, Shochat SJ, Haase GM, Ritchey ML, Kelalis PP, D'Angio GJ (1998). Comparison between single-dose and divided-dose administration of dactinomycin and doxorubicin for patients with Wilms' tumor: a report from the National Wilms' Tumor Study Group. J Clin Oncol.

[B22] Beckwith JB, Palmer NF (1978). Histopathology and prognosis of Wilms tumor. Cancer.

[B23] Breslow NE, Takashima JR, Ritchey ML, Strong LC, Green DM (2000). Renal failure in the Denys-Drash and Wilms tumor-aniridia syndromes. Cancer Res.

[B24] Breslow NE, Takashima JR, Whitton JA, Moksness J, D'Angio GJ, Green DM (1995). Second malignant neoplasms following treatment for Wilms tumor – A report from the National Wilms Tumor Study Group. J Clin Oncol.

[B25] Green DM, Grigoriev YA, Nan B, Takashima JR, Norkool PA, D'Angio GJ, Breslow NE (2001). Congestive heart failure after treatment for Wilms' tumor: a report from the National Wilms' Tumor Study group. J Clin Oncol.

[B26] Green DM, Peabody EM, Nan B, Peterson S, Kalapurakal JA, Breslow NE (2002). Pregnancy outcome after treatment for Wilms tumor: a report from the National Wilms Tumor Study Group. J Clin Oncol.

[B27] National Center for Health Statistics (1999). National Death Index Plus: coded causes of death supplement to the NDI user's manual.

[B28] Greenwood M (1926). The natural duration of cancer. Reports on Public Health and Medical Subjects.

[B29] Index of/~bxc/SPE/library. http://www.biostat.ku.dk/~bxc/SPE/library.

[B30] The R Project for Statistical Computing. http://www.r-project.org/.

[B31] Breslow NE, Day NE (1987). Statistical methods in cancer research, The design and analysis of cohort studies.

[B32] Teplin LA, McClelland GM, Abram KM, Mileusnic D (2005). Early violent death among delinquent youth: a prospective longitudinal study. Pediatrics.

[B33] Patel KV, Eschbach K, Ray LA, Markides KS (2004). Evaluation of mortality data for older Mexican Americans: implications for the Hispanic paradox. Am J Epidemiol.

[B34] Patel KV, Eschbach K, Ray LA, Markides KS (2004). Re: "evaluation of mortality data for older Mexican Americans: implications for the Hispanic paradox" [letter to the editor]. Am J Epidemiol.

